# Learning through mess: Sensemaking visual communication practices in a UK multidisciplinary applied health study

**DOI:** 10.1177/14703572221092410

**Published:** 2022-07-13

**Authors:** Ian Robson

**Affiliations:** Faculty of Health and Life Sciences, Northumbria University, Newcastle-upon-Tyne, UK

**Keywords:** collaboration, interdisciplinary, methods, sensemaking, visual

## Abstract

This article addresses the challenges and opportunities associated with the development of new visual communication practices and outputs, using an example of such work conducted in a UK interdisciplinary applied health project. Reflecting on his role as co-researcher and practice as a visual ethnographer in the study, the author argues that new visual communication practices may emerge from ‘mess’ and even ugliness. In the case discussed, the author comes to terms with mess and elements of failure as potential phenomena of learning through a process of Sensemaking (see Weick’s *Sensemaking in Organizations*, 1995), by applying innovative visual methods to the approach. Through his version of visual Sensemaking, the author identifies a set of principles to inform innovation in collaborative, interdisciplinary visual communication.

## Introduction

Visual communication and visual methods in research have grown in popularity in recent decades (see Barnhurst et al., 2004). For some, they offer the potential to engage and involve audiences, and others hope that visual communication can bridge communication difficulties, especially when diverse groups of researchers and the public work together (Goransson and Fagerholm, 2018). However, visual processes and artefacts used in such research offer no guarantees of engagement, facilitation of enquiry or truthful representation of data. They can just as easily be confusing, exclusive, meaningless, messy or even ugly, and it is not always clear what makes ‘the visual’ useful or not (see Davison et al., 2012). ‘Doing’ visual research is not enough, so methodologists and educators ask that we develop the thinking and process behind methods and techniques. However, we do not often plan what we must learn and, in practice, experience offers the most powerful potential for learning. Learning itself is messy.

I write this reflective article as an educator and researcher with experience of using visual methods to connect, communicate and support collaborative action, utilized here in an interdisciplinary and collaborative health study. I am concerned with visual communication in research because I am a visual ethnographer, that is, someone interested in material enquiry into the way things happen in specific situations. I also write it as someone who had to learn again how to go about visual communication with others and, specifically, how to develop a methodology that could inform future visual interdisciplinary research practice. This article links to the others in this Special Issue through the idea of mess. By mess, I mean the unique status of ‘the visual’ as a form of acting and knowing that is dynamic, ambiguous and polyphonous. In other words, I suggest that visual processes and products do different things for different people. This messy quality, I argue, is both an opportunity for creativity and production of the new, but also can be troublesome if unexamined.

My experience of mess is as a starting point for reflection and action. That the study I discuss here was interdisciplinary was especially significant as it was a new context for my visual practice. The project was a study into the development of multiple long-term health conditions (‘multimorbidity’), which brought together a diverse set of collaborative stakeholders: data scientists, clinical academics, partners in public services and members of the public. I was lead for engagement and impact, and my preference for visual methods in enquiry and visualizing the practice I was part of as a visual ethnographer shaped my contribution. After (collectively) completing the first phase of the study, I began drafting a paper I imagined could be about our ‘successful shared visual language’. Writing prompted me to be explicit about what I was doing with visual communication in the study, but I pressed on without clarity. Having shared an early draft with colleagues, I selectively focused on what I then saw to be their negative feedback:

Why ‘dilemmas’? . . .Again, this sounds verging on the anti-vax . . . I have no idea what it means other than suggesting if I happened to be sat next to the author in the pub I’d move tables . . . I’m completely lost. I don’t know what any of this means but, more to the point, I don’t see why it is relevant . . .This layout is just really confusing . . . No idea what this is . . . I am so lost here that I am only able to scan and not even sure where to start reading anymore – I could be reading Latin.

I saw issues as dilemmas that others may not have seen as such, and saw that the original draft of an article was abstract and did not connect to what my collaborators found productive and useful in working with visual material. This was a troublesome mess because it was unexamined, I reasoned. Anyhow, these responses were my starting point for reflexive insight (Hibbert, 2021) given my new awareness of confusion and frustration: of visual communication that seemed to ‘not be working’. My focus in this article, therefore, is now understanding how visual practices and products, in interdisciplinary contexts, can produce something new and helpful (see Holsanova, 2012), and away from being a troublesome mess, with its exclusion, confusion and dead ends. I therefore write in the first person to connect with my experience, with what worked and what did not, and share what I learnt for my practice as a visual researcher. I write the article having developed and used a new approach to making sense of interdisciplinary visual mess. This emerged as I looked for a way to achieve awareness and reflexivity about the work. Whilst I describe how this emerged and show how it works for me, I use these teachable moments in my experience to inform a broader consideration of ‘what works’ in interdisciplinary research utilizing visual communication for readers.

Before that story is told, some other starting points are needed. I set these out below in relation to (a) visual communication in health domains, and (b) the status of ‘the visual’ as a form of knowledge.

## Literature Review: Images in Health and Philosophy

### On images in health domains

Today, visual communication is a familiar part of primary health provision, research and education. Members of the public, patients, health researchers, educators and clinicians deal with visual communication that relates to illness and health in their daily lives. This is evident in public health discourse (Serlin, 2011) and in the communication of disease (Parrott et al., 2007; Wadhwa, 2009). For instance, many parent-held records include child-growth charts; visual communication is a common part of everyday health informatics and even complex visual phenomena such as images created by Magnetic Resonance Imaging (MRI) scans are familiar to many (Haux, 2010). In patient leaflets, visual elements join with words to form texts (Akrich, 1995), with multiple meanings or functions (Cohen and Moliner, 2021). Indeed, popular reporting of the global Covid-19 pandemic would ‘be literally unimaginable without . . . visual representations’ (Gillman, 2017: xiii).

However, much of this material is underpinned by particular assumptions and worldviews, and is designed to be ‘read’ in particular ways. In discussing visual material in the doctor–patient relationship, Parrott and Kreuter (2011) note the shift from person focus to a focus on the ‘biochemical and pathophysiology of the patient’ as all forms of health communication became dominated by molecular and chemically orientated science as the major paradigm. Within this paradigm, McLaughlin and Clavering (2012) note, visual communication is primarily a tool used by those holding expert status and therefore power. Speaking to established medical paradigms as a context for visual communication, sociologists have urged critical reflection. From their point of view, visual communication is never simply a presentation of facts, even when used in the context of authoritative artefacts such as medical charts. For example, amongst the Visual Analogue Scales (VAS), which are a form of visual psychometric measuring instrument (Klimek et al., 2017), there is one popularly referred to as the Mood Chart, often used as a part of treatment for bipolar disorder (Miklowitz, 2019). Such charts visualize self-reported mood states over time. Martin (2007) asks a specific question about what a mood chart actually measures:

What is the something that goes up and down, or gets a new numerical designation: Moods? Feelings? Energy? Will? Whatever it is, it comes from a private, individual, and interior space. The chart converts specific experiences into obstructions through numeric measurement . . .but it also makes these experiences social along the way. (p. 195)

So, despite being in popular use (Miklowitz, 2019), the work of images in different health domains, as this example indicates, is under-examined. This is not restricted to bipolar disorders; generally, the many lines and charts that illustrate human development in child health records and textbooks show less (or obscure more) than one might expect. For example, Mayer (2009) cites the lack of progress in the visual representations or methods used to show causal linkages and interactions between different variables in human development. As noted, images may be familiar but troublesome.

Elsewhere, in the field of medical humanities and specific fields such as graphic medicine, activity speaks to how research and clinical practice may utilize visual communication differently. Johnstone (2018) proposes that visual medical humanities ‘embraces ambiguity’. Experimental and creative work in the medical humanities expands health discourse, but also informs health practices, as noted in Vaccarella’s (2013: 70) description of how ‘graphic pathographies (book-length comics about illnesses)’ assist medical students’ observation and interpretative skills. That there is so much to be examined may be partly to do with mixing the languages of different traditions and disciplines. Bucher and Niemann (2012: 302), for instance, suggest that ‘scientists, usually more experienced with spoken and written language . . . have to learn how to orchestrate a complex multimodal ensemble of different semiotic systems.’ If a key difficulty is the unexamined consideration and use of visual activities and artefacts, then there is literature to inform this.

### Beyond health: epistemology of the image

It has been a constant belief of scientists, poets and artists alike that an illustration alongside a text is more than just another representation of the same idea. Not only does a picture say more than a thousand words; compared to text, images show different things differently. (Kline, 2014: 1)

As previous examples illustrate, most images relate firmly to words, usually illustrating or framing them. Visual communication differs from the formal symbolic meanings of written communication and so deserves consideration as another category of communication. Visual communication operates as a distinctive category in many ways, especially through how it communicates the phenomena of ambiguity and affect (Crowther, 2021; Gamboni, 2002). It is less reliant on formal symbols and systems of meaning, meaning the rules for ‘reading’ visual communication are less clear, or messier. The implication is that visual communication can be read in multiple ways to mean different things. The more abstract or less familiar it is to the viewer, the greater the potential for ambiguity or polyphony of meaning.

Consideration of what sort of knowledge visual images belong to is an age-old debate. Plato (2007[375 BCE]) had little time for images, questioning how one could represent anything without knowledge of it, and directed knowledge seekers to philosophical discussion. Aristotle (1996[335 BCE]), on the other hand, argued that mimesis (representations of life) could lead to emotional catharsis, or release. Today, disciplines such as psychology have not yet developed a ‘coherent opinion’ on the nature of mental images (Kline, 2014: 4), and the role of the image in thinking ‘has yet to be appreciated’ (Schmidt, 2013: 3). So, debates continue about types of knowledge images contain, especially when involving different disciplines. Some focus on what we can know about the art object itself, as singular interpretations. Others acknowledge that images can elicit an emotional response, whereas others go so far as to say that images (art) can provide information about the world (Novitz, 1998). Even these claims are contested – for example, by arguing that insights produced by fiction do not produce the world as it is. Instead of knowledge of the world, an alternative consideration is that images can develop moral knowledge; we gain access to examples of things we might not otherwise experience, or further, they help us gain imaginative access to relevant insights (John, 2001).

Beyond questions of status, literature can speak to the work that images can do. Here too, philosophers provide different explanations. Gadamer (2013[1975]) took art and aesthetic experience as the reference point for considering the nature of experience and truth. Elsewhere, Foucault (2005[1982]) argued that reflection upon art and aesthetic experience is key to action and transformation. From Greek philosophy onwards, the image (or any ‘work’) is a structure created when a practice is transformed: the image becomes a ‘work’ (p. 21). For hermeneutical philosophers, the image as a ‘work’ has interesting qualities. For Heidegger (2010[1953]), images do work that concepts alone cannot do in that they are both specific examples of things, and things that speak to a general concept. Schmidt (2013: 34) remarks that ‘this doubled, ambiguous nature of the image is at the root of its strangeness.’

One way of considering the work of images is to consider them as interactive experiences. Gadamer (2013[1975]: 22) talks about the ways in which an image discloses something, leading to understanding of a world. This involves something different from the reproduction of reality, but instead of fiction being the opposite of ‘truth’, for Gadamer, it involves a reconstitution of the familiar so that, when viewed in fresh ways, it can be recognized. For Gadamer, this mimesis is not a poor repetition of the world, but is an ‘enlargement’ of it. As an interactive experience, seeing is not a one-way process, but involves feedback from experience and prediction making (Arnheim, 1968). In being seen, the image opens a space of appearance (Schmidt, 2013: 36), or encounter – somewhere work can be done. For hermeneutic philosophers, the image must be read as its own kind of text, but not one measured by scientific standards.

Seen as active, not passive elements, images can be considered to act, or have effect, in different ways. Some of this ‘work’ that visual images can do is generally appreciated. For example, the fact that visual images can operate through metaphor is tacitly recognized. Through metaphor, images can be utilized to transfer meaning from one subject to another (analogy) through juxtaposition, replacement or fusion of images. Hence, one type of ‘work’ that images do is rhetorical – techniques of persuasion that construct a particular meaning. Visual metaphors are arguably a ‘fundamental way of thinking’ (El Refaie, 2019) which relate intimately to our embodied ways of moving and perceiving.

In summary, these examples show that visual communication may do more than reproduce the world or communicate factual content. Some of its potential for both productivity and ‘troublesome mess’ is implied when it is discussed as an interactive event which creates a space of encounter, as metaphor, as opportunity to foreground, or to ‘move’ the viewer in a direct, embodied way. Visual communication has qualities that are open to interpretation and may be experienced in different ways, it can be less precise or fixed than written communication, displaying ambiguity or polyphony (Macleod and Holdridge, 2006). One strand that runs through literature on images and meaning is that images cannot be considered apart from language (Schmidt, 2013: 19). It is inevitable, in life as well as interdisciplinary research, that conscious translations must occur as one moves between word and image, in the knowledge that ‘shifts and alternations’ (p. 68) will occur in such translations. Making use of images in contexts such as health research therefore demands that one finds ways of translating, connecting and relating them to other forms of knowledge so that their ‘messy’ qualities can be utilized to make sense.

## Methodology

Contrasting philosophical claims about the status and potential of images with examples of how they are used in health domains seems to suggest a huge gap. On one hand, images in a medical paradigm mostly exist to illustrate or decorate written, logical argument. On the other, images offer an ambitious but abstract ability to refigure and encounter different perspectives on reality. In the interdisciplinary health study, I wanted to make sense of a mess that seemed troublesome, one in which I was not sure ‘what was going on’ with my visual communication. My goal was practical because I needed to ensure that visual communication was supporting our shared enquiry, so a particular focus for my reflexive work was what was productive, or had potential to be productive in that study’s shared visual activity.

One perspective that offers to connect both the specific/practical seen in health and the speculative and creative potential of images discussed in philosophy is the tradition of Sensemaking. Sensemaking is a term coined by Weick (1995) to describe the process of coming to terms with, and acting from, a situation that is somehow confusing, ambiguous or problematic. In Sensemaking, we ‘make sense’ of ourselves, we reflect, we feel, we connect with others, we pay attention to that which we notice, and we find a practical way forward. Weick presented the seven key features of Sensemaking as: (1) grounded in identity construction; (2) retrospective; (3) enactive of sensible environments; (4) social; (5) ongoing; (6) focused on and by extracted cues; and (7) driven by plausibility rather than accuracy (p. ix). Maitlis and Christianson (2014) identify Sensemaking’s early focus as being concerned with logical–rational tasks of constructing and transmitting meaning, phenomena such as explanation (Starbuck and Millikan, 1988) and cognition (Cornelissen et al., 2010). Calls for Sensemaking research to develop have stressed the need to understand the process of Sensemaking and its ‘pre narrative’ activity (Sandberg and Tsoukas, 2015).

**Figure 1.**
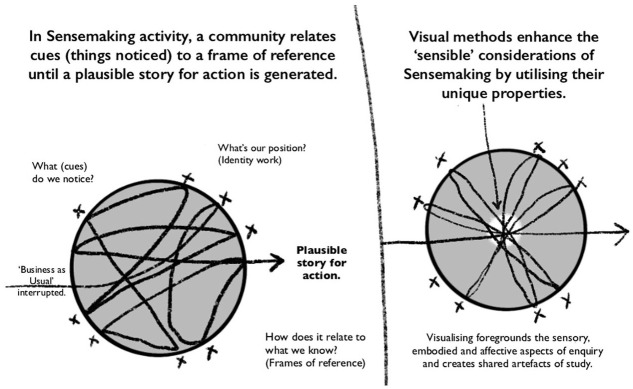
The author’s illustration of the process of Sensemaking, and visual Sensemaking as described in the article.

I was particularly interested in what I saw as the under-developed potential of the third principle listed by Weick (1995), that Sensemaking utilized ‘sensible’ environments. This connected to the philosophical texts I had read about the ability of the visual to ‘do’ work, to enable encounters, and to affect via sensory and material processes. This theme is under-explored in semiotic and Sensemaking literature, but I noted starting points for development. For example, literature recognizes that ‘pre-verbal, pre-conscious, pre-conceptual and pre-intentional processes’ are related to conscious and communicative activity (Salvatore and Freda, 2011: 121). Visuals can affectively ‘move’ those that interact with them, thus acting as an important precursor for cognitive activity. Elsewhere, collective capacity for mindfulness in Sensemaking (e.g. being aware of details, errors in the making and so on) is enabled by what Barry and Meisiek (2010: 1505) call ‘analogous artefacts . . . [things that] induce but do not dictate analogical consideration’ and what Carlile (2002) calls ‘boundary objects’ – artefacts that can cross several domains of knowing. Ultimately, the process of visualizing is recognized as a form of valid enquiry in other traditions such as education (Smith et al., 2015). Therefore, I saw an opportunity to utilize the potential images and imaging in my Sensemaking. If traditional ways of interacting with my data left me confused, I reasoned that visual Sensemaking could expand ways in which I interacted with ‘mess’ through these capacities and more. Figure 1 illustrates the process of Sensemaking as I considered it, and my ambitions for visual Sensemaking.

### Context for the original interdisciplinary collaborative study

As noted, the context for my Sensemaking was as a member of a newly established applied health research collaborative and, specifically, an exploratory study into the developmental origins and mechanisms of multiple long-term health conditions (multimorbidity) as they begin in early life. One aspect of my role as lead for ‘engagement and impact’ was to work with diverse non-clinical and scientific stakeholders as we co-created an understanding of what multimorbidity was, could understand it, explain its key mechanisms and possible points of intervention for health and other practitioners. As a visual researcher, I had earlier found that visualizing illness was a potentially contentious matter. For example, in the work I led with parent and carer representatives, supported by a UK children’s charity, we found that visually communicating the development of illness across the life course raised difficult questions (e.g. in the relative emphasis on contextual factors or lifestyle choices in health, or the extent to which a focus on illness development was deterministic and pathological). The practical activities involved in the multimorbidity study included the production of visual consultation materials for parent and patient stakeholder groups, creation of visuals for events and presentations, and development of an interactive ‘causal map’ of multimorbidity. In addition, much of my personal correspondence with fellow researchers utilized visual note-making. In the light of feedback, I had to work out why I now found this to be a ‘mess’, and find what, if anything, was productive in it.

**Figure 2.**
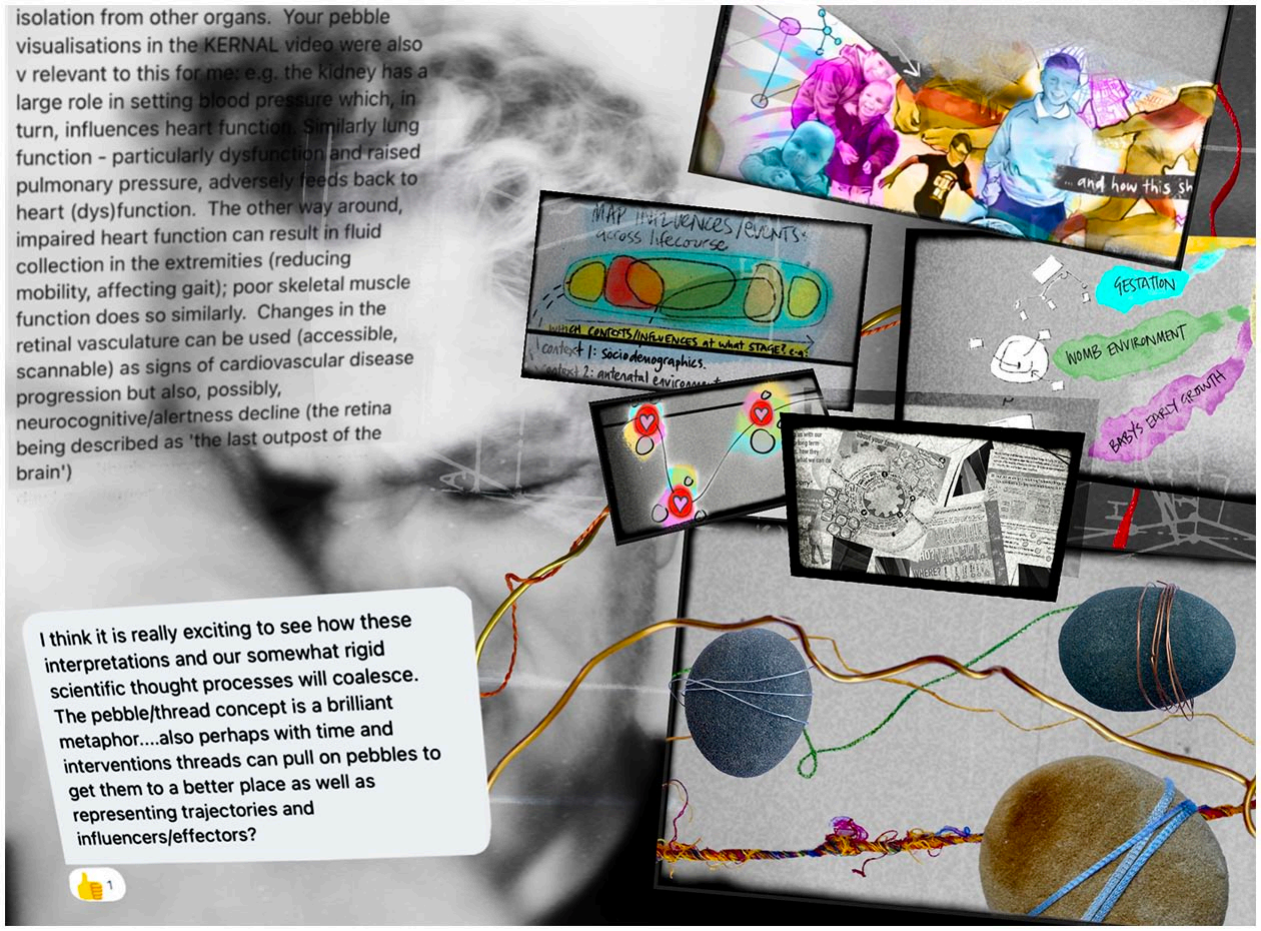
An image presenting a range of messy ‘starting points’ for Sensemaking after the study had completed.

**Figure 3.**
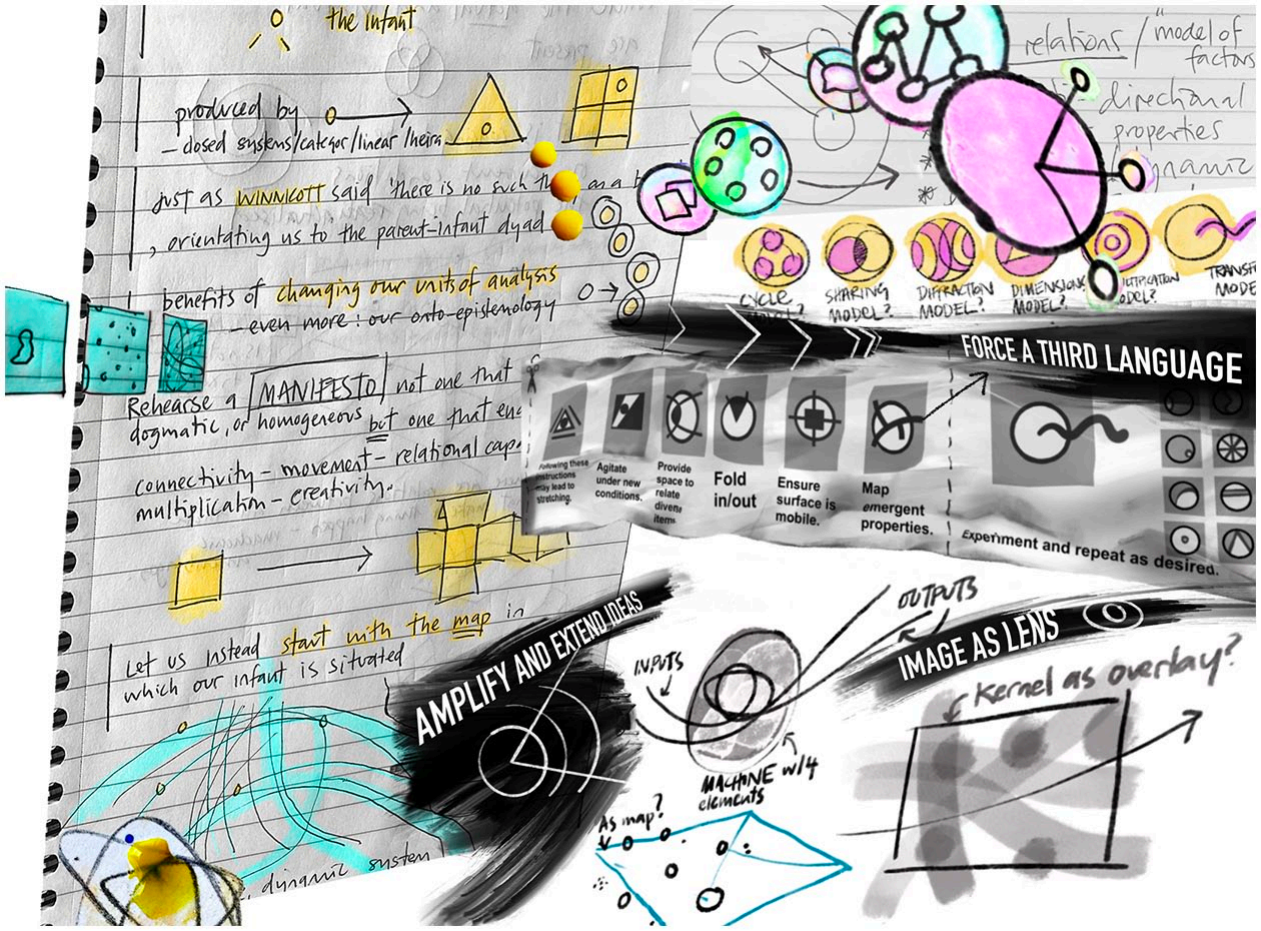
A collage of visual notes created and used in dialogue with the author’s collaborators in the study of multimorbidity.

As part of this diverse group, I was required to collaborate at a fast pace during an initial exploratory phase of the study. Members of the study team related well socially, but had different communication styles and disciplinary perspectives. Working with those who were different from me was rewarding, but I took time to remember that our respective appreciations and uses of visual communication were based on different paradigms (see Table 1), different systems of knowing about and acting upon the world (Kuhn, 2012[1962]). Initially, most of my experiences in using visual communication seemed productive – on a day-to-day basis, I would create sketch-notes in advance, or following meetings, and these visual notes provoked discussion and provided a starting point for shared enquiry (Figure 3). As might be expected, as the study progressed and I worked with others to develop more formal visual representations of multimorbidity, things became more challenging. Towards the end of the study, I was delighted that visual notes provoked new lines of enquiry (for example, when artistic photography of stones and threads could help us talk about the interconnected nature of human development) but I was also disappointed when progress was slow in producing a causal map of multimorbidity, or when my presentation materials seemed little more than decorative. If I was infuriated, as well as enthused, something must have mattered, I reasoned. Practically, I felt already in the middle of this mess, so there was no neat moment when I reviewed the data. My experience of Sensemaking felt removed from any simple description of Weick’s (1995) process being a rational, retrospective identification of cues, assessed against a single frame of reference. Creating images (e.g. Figure 2) provided a method which would bring together temporal events, perspectives and artefacts, enabling non-linear and more-than-rational work with my messy data.

**Table 1.** The author’s view of example collaborators’ paradigms and implications for activity with visual communication.

Example Collaborator Group	My perception of what characterised their paradigm	How I understood their orientation to visual communication in the study.
Visual ethnographer (author)	Heuristic, creative, reflexive, relational.	As Sensemaking process, connector, dialogue.
Parent Volunteers	Experiential, practical, reflective, ethical focus.	As tool, topic information, site of enquiry.
Clinical Academic and Scientific Academic Colleagues	Objective, analytical, logical.	As decoration, discussion starter, external consultation tool.

**Figure 4.**
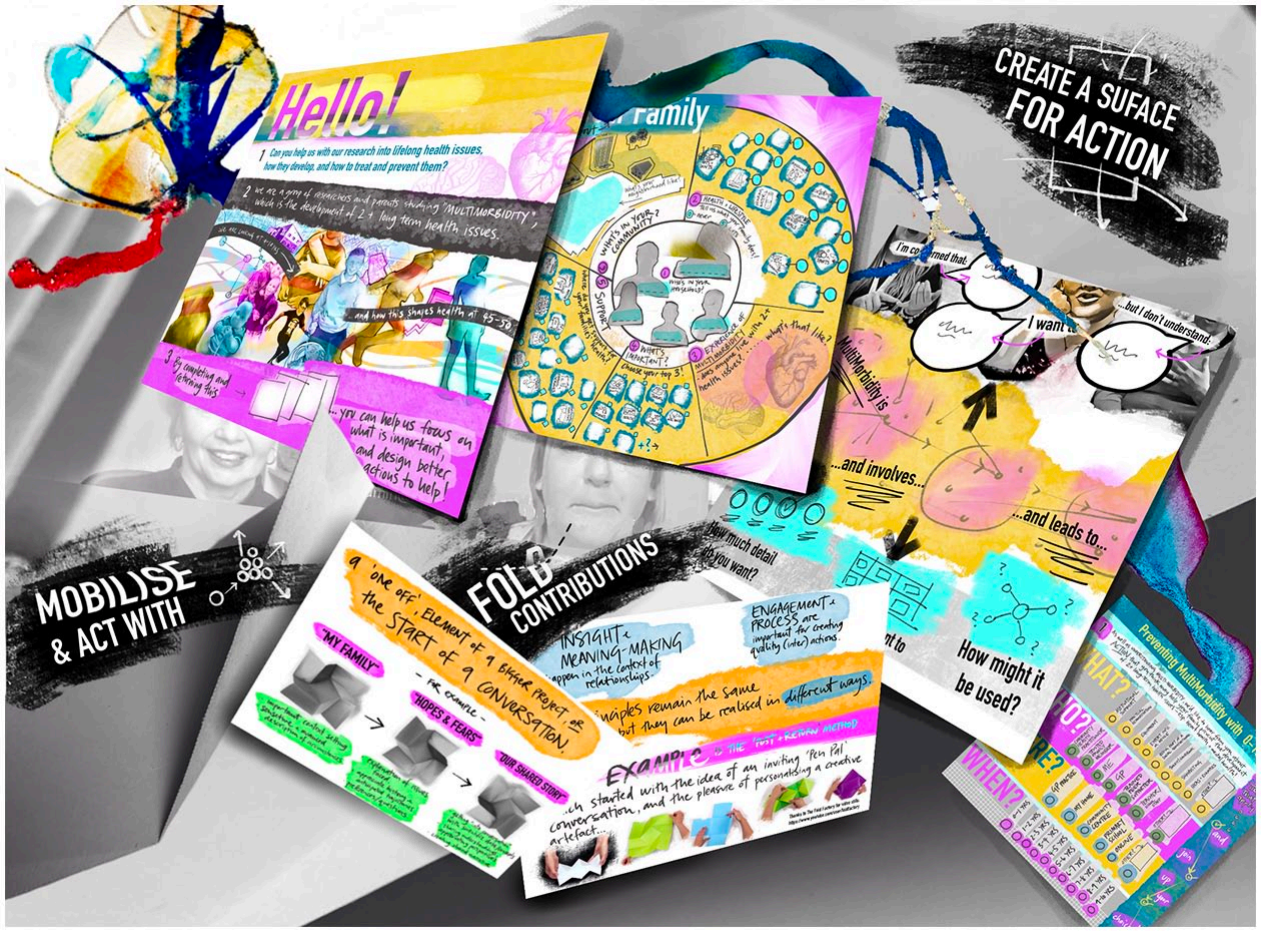
An image which reanimates the process of creating a consultation prototype with parent-volunteers.

**Figure 5.**
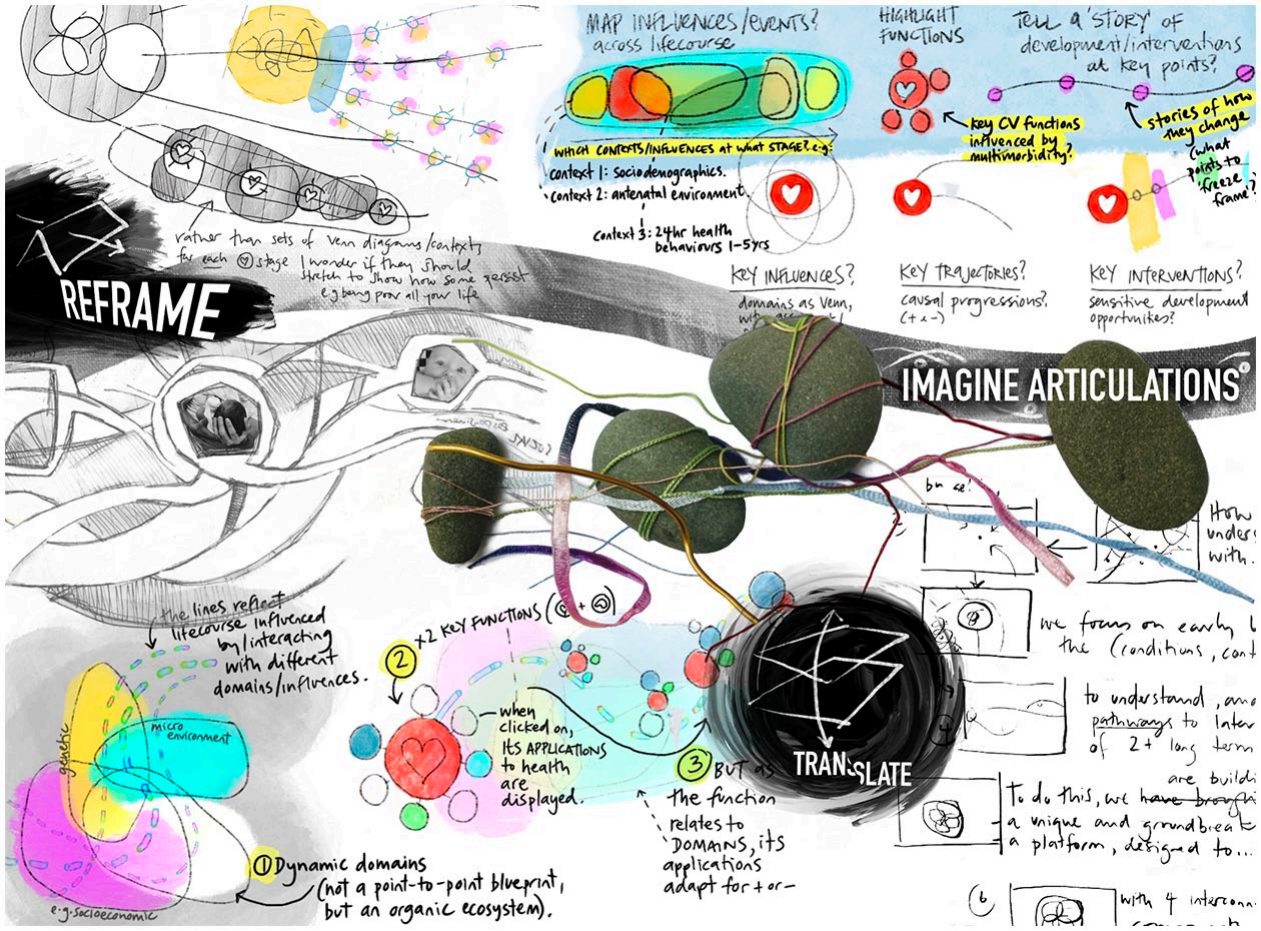
Visual elements relating to the construction of a ‘causal map’ of multimorbidity in the study.

Challenging feedback mentioned earlier led me to review all the data (visual material, notes and correspondence) created in and for the study, to notice cues, those things that stood out as strange, interesting or irritating, that could act as starting points for enquiry (Weick, 1995), including moments that may have been productive. From this point, I focused my Sensemaking on three aspects of the work that seemed to be clear categories: (a) informal visual communication in the form of visual notes; (b) work with parent-volunteers to create consultation materials; and (c) a causal map of multimorbidity. At the same time, I also named some of the theoretical frames of reference as, until I acknowledged them, I found I was unable to make progress just as Weick’s (1995) principles of Sensemaking make clear. As I considered ‘the visual’ as potentially ambiguous, sensory and polyphonous event, following Gadamer (2013[1975]) and others, my philosophical frame of reference encouraged me to consider all the different types of data I had about visual communication in the study, including notes, sketches, emotions, products and correspondence. I needed a basis on which to select this data, and a basis on which to bring it together in a Sensemaking task. Practically, I decided to create a set of images (Figures 3, 4 and 5) that would allow me to encounter data again. Doing this allowed me to expand Weick’s (1995) insight that Sensemaking was a ‘social’ process between people: refiguring and materializing data in Sensemaking images brought me into a social relationship with images themselves. I discovered that my Sensemaking images had an agency and affective power of their own, with the ability to present themselves (Marion, 2003). As images enabled data to become social, I could attune to ways in which visual elements ‘glowed’ (Maclure, 2010, 2013b) and produced a sense of wonder, as discussed by Maclure (2013b: 228–229):

I think we need more wonder in qualitative research, and especially in our engagements with data, as a counterpart to the exercise of reason through interpretation, classification, and representation . . . Wonder is not necessarily a safe, comforting, or uncomplicatedly positive affect. It shades into curiosity, horror, fascination, disgust, and monstrosity.

I imported images from my study archive into a digital illustration software program, based on the criteria of what seemed to generate this sense of wonder. Working with these selected marks, images and clippings of text, and adding to them, constituted the visual sensemaking I was looking for and had previously practiced (Robson, 2021). As elements were montaged in a digital software tool (positioned, cropped, drawn upon and so on), a set of Sensemaking images (Figures 3, 4 and 5) was created. Once I was able to materialize some of the visual elements, I could direct enquiry about what was productive or had the potential to be productive, using my bodily sensations (i.e. between the image and scanning eye, drawing hand and quickening heartbeat). I saw this as a material, embodied and affective form of Sensemaking (Weick, 1995) and sensory (auto)ethnography (Pink, 2015) which foregrounded key sensations, ideas or concepts. Figure 3 dealt with assorted visual note-making in the study, Figure 4 dealt with work I had done with parent-volunteers to create a prototype consultation booklet and Figure 5 dealt with the development of a causal map of life course health and illness.

## Findings and Reflections

In this section, I consider key insights produced from my visual Sensemaking activity, but I first return to the context of the study to consider the issue of ‘audience’. My position, as one who undertook the visual Sensemaking, was as a member of a research collaborative that intentionally connected to a range of stakeholders – the wider early child health research community, the research collaborative members I had undertaken research with, and our consultation partners who were asked to make their own sense of the topic of multimorbidity. Insights that follow therefore are potentially applicable to all of these stakeholder groups, as our ‘research’ was applied in nature, as we developed, refined and tested concepts and findings in practice and service user contexts. At the level of principle, what ‘worked’ in the collaborative speaks to what could work in visual consultation, and indeed in wider efforts to generate and interact with data in the child health research community. Each can take these principles and engage in their own Sensemaking as they apply them to their visual communication.

### Making sense of visual notes

In Figure 3, I created an image that focused on visual notes I made in the study, most often created within a dialogue with others. There are frequent uses of frames and symbols in the image, perhaps because these spoke to how phenomena such as child health data could be structured and questions of what was happening in the development of life course illness. Lines, boxes and visual icons seemingly struggled to contain activity. Once I had composed, drawn and digitally pasted into the image, three annotations (for practical purposes, the ‘results’ of the Sensemaking activity) captured the activity I sensed:

‘*Amplify and extend ideas*’: I saw that visual communication helped me to materialize emerging thoughts, ideas and feelings from correspondence or interactions with colleagues. I would often seize on a common idea, image or analogy and take the opportunity to extend this: (a) by making it more concrete and explicit, and (b) by imagining how it might be used in presenting further ideas.

‘*Force a third language*’: In the original activity, and this new Sensemaking activity, I used visual communication to create a parallel ‘what if’ conversation. This had the effect of disturbing existing lines of argument and provoking us to contribute to a new event of making sense, instead of repeating existing positions.

‘*Image as lens*’: Once used in interactions, elements in visual notes supported play and experimentation. A suggested function could be applied to an example, or a question could be posed, such as ‘in what way do those things work together?’.

### Making sense of a consultation prototype

Figure 4 derived from a process where I worked with a parent-volunteer subgroup to co-design a prototype booklet for prospective consultation audiences on this unfamiliar idea of multimorbidity. Multiple pages depict the constant revisions made in cycles of making, sharing, discussing and re-making. Clutter and excess detail imply the challenges faced in connecting issues and questions. Images float on photographs of folded paper and imagined encounters between consultees and the booklet. In the image, folding became a metaphor for contributions, and the necessary discomfort of willingly bringing contributions to see them change, or get lost, in collective work. Again, I noted three activities that had held my attention as I made the new composite image:

‘*Create a surface for action*’: elements of visual communication became a surface for connecting separate observations, insights and questions (e.g. ‘what if this came next?’; or ‘would it be better if . . .?’). We could work on what we could see, each ‘reading’ the image to see how or if it worked. Activity did not produce a workable prototype, but the image supported critical review and suggestions.

‘*Fold contributions*’: when the group interacted with sketches and versions of prototype pages, productive work seemed to be driven by moments of enthusiasm or displeasure. The folding of contributions always produced a new variation of the layouts which was energized by the reception of, and work with, each version of the prototype.

‘*Mobilize and act with*’: the image was not enough. I remembered how we printed a final draft for testing with the groups’ family, friends and neighbours. Materializing the designs created artefacts and transformed parent-volunteers into presenters. Changing the format and interaction with the prototype transformed the work it could do.

### Making sense of an interactive map of life course health and illness

One of the most challenging processes was the development of a ‘causal map’, which showed how multimorbidity developed in early life and onwards, informed by a detailed literature search for evidence on early life determinants of later life multimorbidity. My attempts to visualize the results confirmed the views of the team who had completed the literature search that creating a ‘causal map’ would be problematic. This was largely because the studies found were very different from one another with few elements to link them . . . what was described did not speak to linkages between elements, or cumulative effects. Visual Sensemaking helped me remember cycles of frustration, which involved asking ‘what do you want me to visualize again?’ When I showed the parent-volunteer group simple sketches abstracting the data, they just found them confusing, not in terms of scientific assessment, but because they did not know how to read or use them. Their analysis of very early versions of the map immediately threw up pained faces over video calls, with questions like ‘what is this for?’, ‘it’s a depressing story’, ‘so it’s all my fault?’, ‘what difference does it make?’ and so on. Visualizing foregrounded questions – were we creating a fatalistic tale of individual moral failures, as if illness was just about ‘laziness’, or ‘gluttony’? In such a map, where was the agency to challenge and change structural inequalities linked to the development of illness?

Whilst wondering if more data would come, I dealt with our frustration by sketching flows (see middle left-hand side, Figure 5) to materialize imagined movement of health and illness across the life course. This threw up more questions: ‘what is it that flows?’ and (in respect to the ethical discomfort) ‘what’s currently in the background that needs visualizing?’ Using the visuals helped me imagine the ‘line’ of life course health as dynamic movement (see top-right corner and bottom-left corner, Figure 5), speculating, for example, whether a health trajectory would be curved as it passed through what I labelled ‘contexts/influences’ such as relative poverty.

Moving and composing in the image (Figure 5) distilled the following Sensemaking statements:

‘*Imagine articulations*’: Visual elements materialize a model to test: what leads to what? What relations are we talking about? Do we talk about flows? Where do we pan and zoom?

‘*Reframe*’: Experimentation involves asking (and testing) ‘what else can this be?’, taking the topic and pushing back against the representation of multimorbidity as single left to right line which seems to individualize illness. Things (bubbles, lines, other points) float beneath the line, challenging the representation of developing multimorbidity as the sole result of ‘poor’ individual choices.

‘*Translate*’: as ideas and dialogue are materialized as visual communication, tacit assumptions and potentials appear. Authors can ask ‘is that how you see it?’ When drawn as a sketch, ideas can be annotated and added to. When the thinking is seen, it becomes possible to interrogate the images, with questions like ‘but what is being collected as the heart icon moves down the line?’

## Conclusion

Mess is necessary in interdisciplinary, collaborative and developmental work, such as the example in this article. To resist mess is to resist enquiry, learning and innovation, but we are encouraged instead to foreground the perfect, the impressive and convincing. Mess can be suppressed but, in doing so, we remove a huge resource for learning. Many artists and educators embrace mess in visual communication, describing visual thinking as an emerging process (Sousanis, 2017). In my experience described here, I have argued that one of the resources for addressing mess and for learning from what is and what is not ‘working’ comes from the Sensemaking tradition (Weick, 1995). Sensemaking starts with the problem as event, discomfort or question and forces the practice of social questioning, seeking for cues, utilizing frames of reference and considering what a feasible solution could look like. However, it is not ideally adapted or applied to visual communication and its processes. The cognitive orientation that has historically been part of Sensemaking (Maitlis and Christianson, 2014) has privileged certain sorts of activity, including rational review and narrative articulation of meaning at the expense of meaning-making itself. In seeking to undertake Sensemaking of visual practices and artefacts, my experience was that I needed to be able to correspond with the visual, enabling translations across images and words, so that I could better surface issues with others, so improving interdisciplinary collaborative research.

My creation of collage-like Sensemaking images is an example of a Sensemaking better equipped to correspond with the visual, a method which supported dialogues between images, the senses and emotions and rational dialogue. This visual Sensemaking method is one that visual practitioners could adapt and develop new approaches suited to their task. The method ‘worked’ in this case because it extended Weick’s (1995) appreciation that Sensemaking was also sensory, embodied and emotional (i.e. ‘enactive of sensible environments’), and utilized the unique epistemological properties of the visual – which include its ambiguity, affective capacity and polyphonous nature.

Sensemaking is consideration of things together – but I have shown that, in this collaborative research, visual Sensemaking must literally bring things together in the frame. Materializing diverse elements in a series of images enables visual Sensemaking work to be done, sifting what is troublesome and productive about mess. In visual Sensemaking, our aesthetic and sensory faculties are utilized to greater effect. As we compose images, we read for patterns, test relations between elements, and more. Visualizing the process of Sensemaking materializes the process, and enables social aspects of the practice. This has implications for visual practitioners seeking to work in reflexive, interdisciplinary and collaborative ways. Firstly, following Gadamer (2913[1975]) to see visual Sensemaking as event(s) in the process of relational dialogue, with different parties being aware of, and choosing to be part of that event. In other words, visual Sensemaking is not ‘automatic’ and the inclusion of ‘the visual’ offers no guarantees of additional insight or benefit. Secondly, that those who would be part of the dialogue of visual Sensemaking can learn to attend to the sensory, embodied and affective aspects visual communication can present, as I did. In doing this, the benefits of using visual processes and artefacts are realized – we learn to have different conversations, and appreciate different aspects of phenomena. Thirdly, practitioners must be committed to the reflexive (Hibbert, 2021) demands of visual Sensemaking: to appreciate when visuals resonate or clash with other ways of knowing, and why that might be. Visual Sensemaking therefore demands that we learn to ‘hold the mess’ and see perspective change as a useful tool. Finally, the sort of ‘work’ involved in sifting productive from troublesome mess must be recognized. As I found, practitioners should be ready to ‘put in the (cognitive, emotional) work’ to translate across boundaries between text and image, and disciplines. Without the appreciation of visual Sensemaking as both an art and science, I suggest it will be difficult to learn to work with mess and to enable productive dialogue in a visual world.
